# Relationships between Socioeconomic Status, Handgrip Strength, and Non-Alcoholic Fatty Liver Disease in Middle-Aged Adults

**DOI:** 10.3390/ijerph18041892

**Published:** 2021-02-16

**Authors:** Jinkyung Cho, Inhwan Lee, Dong-Ho Park, Hyo-Bum Kwak, Kisuk Min

**Affiliations:** 1 Institute of Sports & Arts Convergence (ISAC), Inha University, Incheon 22212, Korea; chojk@kspo.or.kr (J.C.); dparkosu@inha.ac.kr (D.-H.P.); kwakhb@inha.ac.kr (H.-B.K.); 2 Department of Sport Science, Korea Institute of Sport Science, Seoul 01794, Korea; 3 Department of Sports Science, Sungkyunkwan University, Suwon 16419, Korea; ansh00@skku.edu; 4 Program in Biomedical Science and Engineering, Department of Kinesiology, Inha University, Incheon 22212, Korea; 5 Department of Kinesiology, College of Health Sciences, University of Texas at El Paso, El Paso, TX 79968, USA

**Keywords:** socioeconomic status, handgrip strength, non-alcoholic fatty liver disease

## Abstract

Although low socioeconomic status (SES) and decreased muscle strength have been found to be associated with the risk factors of non-alcoholic fatty liver disease (NAFLD), including insulin resistance, obesity, and metabolic syndrome, the associations among SES, muscle strength, and NAFLD are still unclear. We aimed to investigate the combined effect of SES and relative handgrip strength (HGS) on the risk of NAFLD in middle-aged adults. Data from 5272 middle-aged adults who participated in the Korea National Health and Nutrition Examination Surveys (KNHANES) from 2014–2018 were analyzed. NAFLD was defined using the hepatic steatosis index (HSI) > 36 and the comprehensive NAFLD score (CNS) ≥ 40 in the absence of other causes of liver disease. SES was based on a self-reported questionnaire. Overall, individuals with low SES (odds ratio (OR) = 1.703, 95% confidence interval (CI): 1.424–2.037, *p* < 0.001) or low HGS (OR = 12.161, 95% CI: 9.548–15.488, *p* < 0.001) had a significantly higher risk of NAFLD. The joint association analysis showed that a low SES combined with a low HGS (OR = 2.479, 95% CI: 1.351–4.549, *p* = 0.003) further significantly increased the risk of NAFLD when adjusted for all the covariates, compared with individuals with a high SES and a high HGS (OR = 1). The current findings suggest that both low SES and low HGS were independently and synergistically associated with an increased risk of NAFLD in middle-aged Korean adults.

## 1. Introduction

Non-alcoholic fatty liver disease (NAFLD) is the most common type of liver disease, with a global prevalence exceeding approximately 25% [1]. In addition, approximately 30% of individuals are affected by NAFLD in the United States, and approximately 5% of individuals suffer from severe cases of NAFLD, including steatohepatitis and cirrhosis [2]. In Asia, over 27% of individuals are diagnosed with NAFLD [1]. Therefore, NAFLD is not only a global healthcare problem [3], but also a major indicator of mortality [3,4]. The risk factors of NAFLD vary according to age, metabolic syndrome (diabetes, hypertension, obesity, and hyperlipidemia), and diet [5]. Most of these risk factors are modifiable; therefore, clinicians emphasize the importance of aggressive lifestyle changes to improve NAFLD with physical activity, healthy diet, and weight loss. In addition, socioeconomic status (SES) is emergently thought to be an influencing factor for NAFLD [6,7]. Though the reason for SES being included as a risk factor for NAFLD has not been clearly explained, it may closely affect individual lifestyles and living environments. SES disparities can influence dietary habits, accessibility to the healthcare system, and interest, time, and allostatic conditions for exercise [8,9,10]. Because of these possible reasons, SES may be related to the major risk factors of NAFLD, including insulin resistance, obesity, and lipid metabolic disorder [6]. However, there is a conflicting report that SES does not impact the development of NAFLD [11]. Therefore, further studies are needed to determine the relationship between SES and NAFLD, especially in middle-aged adults. Generally, handgrip strength (HGS) is often considered as an indicator of muscle mass and strength. Since HGS is closely associated with body mass [12], relative HGS, which is calculated as absolute HGS divided by body mass index (BMI), has been recommended to measure muscle health in public health and in clinical practice [13,14]. According to previous studies, muscular strength appears to be inversely related to insulin sensitivity [15], dyslipidemia [16], and excessive body and abdominal fat [17], which are independent risk factors for developing NAFLD. Furthermore, growing evidence suggests that low levels of HGS are significantly related to an increased prevalence [18] and severity [19] of NAFLD. In addition, HGS is positively associated with SES components such as education [20], household income [21], and wealth [22]. Individuals with a low SES are associated with reduced access to adequate nutrition, which may partly reflect as declines in muscle mass and strength. However, there is inadequate understanding of the interaction between HGS and SES with regard to NAFLD. Thus, the purpose of the present study was to investigate the effects of SES and HGS on the risk of NAFLD using a large national representative sample of a middle-aged Korean population.

## 2. Materials and Methods

### 2.1. Participants

**Figure 1:**
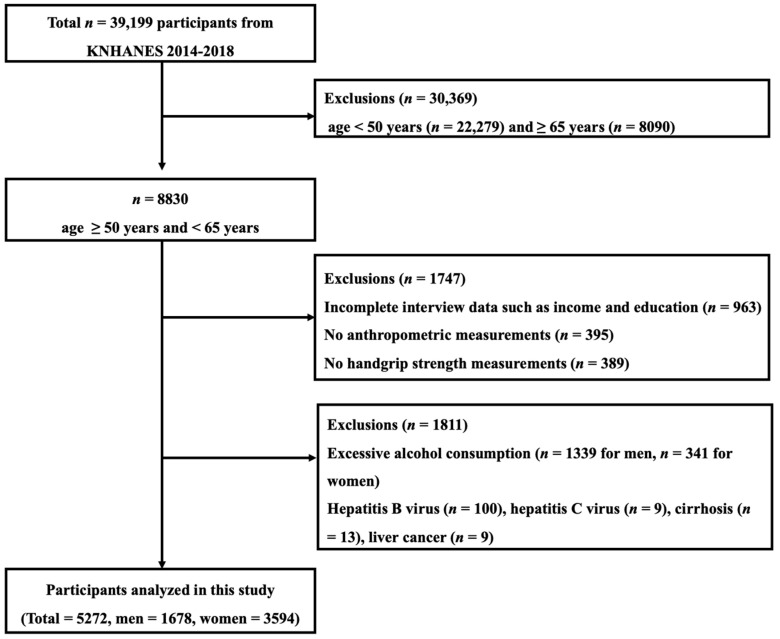
Schematic representation of the participant selection process.

In this cross-sectional study, the National Health and Nutrition Examination Surveys (KNHANES) data from 2014 to 2018 were used. Figure 1 shows the schematic procedure of participant selection. Of the 39,199 individuals, we excluded subjects who met the following criteria: (1) age < 50 years (*n* = 22,279) and ≥65 years (*n* = 8090); (2) absence of a questionnaire on income and/or education (*n* = 963); (3) absence of grip-strength data (*n* = 389); (4) alcohol consumption per week > 140 g for men (*n* = 1339) and >70 g for women (*n* = 341); (5) absence of anthropometric measurements (*n* = 395); and (6) having hepatitis B virus (*n* = 100) or hepatitis C virus (*n* = 9) infections, and having cirrhosis (*n* = 13) or liver cancer (*n* = 9). Finally, 5272 middle-aged adults participated in this study.

### 2.2. Socioeconomic Status

Income and education based on a self-reported questionnaire were used as socioeconomic indicators. Income was divided into quintiles, and education level was calculated by the total number of years. For a composite score for SES, the determinants of education and income were multiplied to create combined scores ranging from to 0–100, with higher values representing a high SES [23]. The SES index was divided into quartiles and defined as low SES (<25th percentile), middle SES (25th–74th percentile), and high SES (≥75th percentile).

### 2.3. Covariates

All the participants were examined for anthropometric data, sociodemographic status, health-related factors, and blood markers. Anthropometric data included age, BMI, and waist circumference (WC). BMI was calculated by dividing the body weight by the square of the height (kg/m^2^). WC was measured based on the midpoint of the lower rib and upper iliac ridge. The sociodemographic status indicators, including marital status (married, widow, or divorced and unmarried), region (urban or rural), and type of housing (apartment or general house) were used as covariates. Health-related factors such as smoking, alcohol consumption, regular exercise, hypertension, diabetes, and menopause were included. Smokers were defined as current smokers or ex-smokers who had smoked over 5 packs of cigarettes in their lifetimes. Alcohol consumption was defined as those who consumed alcohol more than once a week. Subjects were categorized as regular exercise if they performed either moderate or vigorous intensity exercise for at least 30 min at a time and at least twice per week. Hypertension was defined as blood pressure ≥ 130/85 mmHg or by the usage of antihypertensive medications. Diabetes was defined as fasting glucose levels ≥ 126 mg/dL or by the diagnosis of a physician. Fasting blood glucose (FBG), high-density lipoprotein cholesterol (HDL-C), triglyceride (TG), aspartate aminotransferase (AST), alanine aminotransferase (ALT), and uric acid levels were measured using the Hitachi Automatic Analyzer 7600-210 (Hitachi, Tokyo, Japan).

### 2.4. Definition of NAFLD

NAFLD was defined using the hepatic steatosis index (HSI) and the comprehensive NAFLD score (CNS) [24]. HSI and CNS have been validated in previous studies to define and predict NAFLD in the Korean population [25,26]. HSI was calculated as follows: 8 (alanine transaminase (ALT)/aspartate transaminase (AST)) ratio + BMI (+2 if diabetes mellitus (DM) status + 2 if female). Values > 36 were considered to indicate NAFLD. CNS was calculated as follows: probability (in %) of having NAFLD = 1/(1 + exp(−x)) ×100. If male, x = 0.016 × age (years) + 0.182 × BMI (kg/m^2^) + 0.089 × WC (cm) + 0.391 × alcohol (yes = 1, no = 0) + 0.124 × exercise (yes = 1, no = 0) + 0.018 × fasting glucose (mg/dL) + 0.773 × log_e_ (triglycerides (mg/dL)) − 0.014 × HDL cholesterol (mg/dL) + 0.145 × uric acid (mg/dL) − 0.674 × log_e_ (AST (IU/L)) + 1.632 × log_e_ (ALT (IU/L)) −21.695. If female, x = 0.320 × BMI (kg/m^2^) + 0.044 × WC (cm) 0.533 × diabetes (yes = 1, no = 0) + 0.016 × fasting glucose (mg/dL) + 0.951 × log_e_ (triglycerides (mg/dL)) − 0.015 × HDL cholesterol (mg/dL) + 0.199 × uric acid (mg/dL) − 0.645 × log_e_ (AST (IU/L)) + 1.302 × log_e_ (ALT (IU/L)) + 0.255 × menopause (yes = 1, no = 0) − 19.741. Values ≥ 40 were considered to indicate NAFLD.

### 2.5. Relative Handgrip Strength

The handgrip strengths (HGSs) were measured three times using a handgrip dynamometer (Digital Grip Dynamometer, TKK 5401, Takei Scientific Instruments Co., Ltd., Tokyo, Japan). The maximum value among the measured values was considered as the absolute HGS. The relative HGS was calculated as absolute HGS divided by BMI. The relative HGSs were divided into gender-specific quartiles and categorized as low HGS (<25th percentile), middle HGS (25th–74th percentile), and high HGS (≥75th percentile).

### 2.6. Statistics

Statistical analysis was performed using Statistical Package for the Social Sciences for Windows, version 24.0 (SPSS Inc., Chicago, IL, USA). All the variables were checked for normality, and if needed, a log10 transformation was conducted before the analysis. *p*-values < 0.05 were considered statistically significant. Continuous variables were presented as means and standard deviations. Descriptive analyses of sociodemographic status and health-related factors were conducted using proportions (%) for categorical variables. AN independent t-test and a one-way ANOVA were performed to compare the mean differences according to gender, SES, and HGS subgroups. Binary logistic regression analysis was conducted to estimate the risk (odds ratio (OR)) of NAFLD for the combination of SES and HGS after adjusting for age, WC, marital status, region, type of housing, smoking, alcohol consumption, regular exercise, hypertension, and menopause. High SES with high HGS was used as the reference group (OR = 1).

## 3. Results

Table 1 summarizes the baseline characteristics of the 5272 study participants based on gender. The group of men had subjects with higher BMI (*p* < 0.001), WC (*p* < 0.001), absolute HGS (*p* < 0.001), relative HGS (*p* < 0.001), household incomes (*p* < 0.001), educations (*p* < 0.001), and number of participants with the married status (*p* < 0.001) than women. A higher percentage of women lived in urban areas than men (*p* = 0.009). With respect to health-related factors, men had higher percentages of smoking (*p* < 0.001), alcohol consumption (*p* < 0.001), regular exercise (*p* < 0.001), hypertension (*p* = 0.002), and diabetes (*p* < 0.001) than women.

Table 2 shows the descriptive statistics for the measured parameters of the three subgroups based on the SES. The low-SES group had subjects with higher ages (*p* < 0.001), BMI (*p* < 0.001), and WC (*p* < 0.001), and greater proportions of smokers (*p* < 0.001) and those with hypertension (*p* < 0.001) and diabetes (*p* < 0.001) compared to the high-SES group. On the other hand, the high-SES group had a higher relative HGS (*p* < 0.001), as well as proportions of those with married status (*p* < 0.001) and those living in urban areas (*p* < 0.001) and apartments (*p* < 0.001) than the low-SES group. Subjects with a low SES had higher FBG (*p* < 0.001), TG (*p* < 0.001), AST (*p* < 0.001), and ALT (*p* = 0.011) levels, whereas subjects with a high SES had higher HDL-C (*p* < 0.001) and regular exercise (*p* < 0.001). NAFLD indices, including HSI (*p* < 0.001) and CNS (*p* < 0.001) were the highest in the low-SES group, followed by the middle-SES and high-SES groups.

Table 3 presents the descriptive statistics for the measured parameters of the three subgroups according to the relative HGS status. The subjects with a high HGS had higher household incomes (*p* < 0.001), educations (*p* < 0.001), and SES indices (*p* < 0.001) than those with a low HGS. The low-HGS group had subjects with a higher age (*p* < 0.001), BMI (*p* < 0.001), and WC (*p* < 0.001), and those with hypertension (*p* < 0.001), diabetes (*p* < 0.001), and menopause (*p* = 0.028), but not regular exercise (*p* < 0.001) than the high-HGS group. The high-HGS group had a higher percentage of those with married status (*p* < 0.001) and those living in apartments (*p* < 0.001) than the low-HGS group. With respect to blood markers, the low-HGS group had higher FBG (*p* < 0.001), TG (*p* < 0.001), AST (*p* < 0.001), and ALT (*p* < 0.001) levels than the high-HGS group. On the other hand, the high-HGS group had higher HDL-C (*p* < 0.001) and uric acid (*p* < 0.001) levels than the low HGS group. Furthermore, NAFLD indices including HSI (*p* < 0.001) and CNS (*p* < 0.001) were significantly different among the three subgroups. The mean HSI and CNS scores were the highest in the low-HGS group, followed by the middle-HGS and high-HGS groups.

In the 5272 subjects, the prevalence of NAFLD based on HSI and CNS scores was 25.1% and 58.8%, respectively. Regarding the prevalence of NAFLD in Korea, we estimated the risk of NAFLD using the HSI score [23]. Using binary logistic regression, the ORs for the risk of NAFLD according to SES and relative HGS status were estimated (Table 4). With respect to SES, the low-SES (OR = 1.703, 95% CI = 1.424–2.037, *p* < 0.001) and middle-SES groups (OR = 1.340, 95% CI = 1.144–1.570, *p* < 0.001) had significantly higher risks of developing NAFLD than the high-SES group (reference, OR = 1). Furthermore, for each one-point increase in SES, the ORs for having steatosis significantly increased by 1.008 (95% CI = 1.006–1.011, *p* < 0.001).

With respect to relative HGS, the low-HGS (OR = 12.161, 95% CI = 9.548–15.488, *p* < 0.001) and middle-HGS groups (OR = 4.300, 95% CI = 3.402–5.435, *p* < 0.001) had significantly higher risks of developing NAFLD compared to the high-HGS group (reference, OR = 1). For each 0.1 kg/BMI in HGS, the OR for having NAFLD significantly increased by 1.157 (95% CI = 1.135–1.179, *p* < 0.001).

Lastly, we investigated the joint association of SES and relative HGS with the risk of NAFLD. Compared with high SES plus high HGS as a reference (OR = 1), high SES plus low HGS (OR = 9.286, 95% CI = 5.868–14.694, *p* < 0.001), and low SES plus low HGS (OR = 13.499, 95% CI = 8.755–20.812, *p* < 0.001) had significantly higher risks of NAFLD (Table 5). This association remained statistically significant even after adjusting for several covariates, including age, WC, marital status, region, type of housing, smoking, alcohol consumption, regular exercise, hypertension, and menopause.

## 4. Discussion

In this population-based prospective study, we studied the independent and joint effects of SES and HGS on the risk of NAFLD in middle-aged Korean adults. Our results demonstrated that a low SES or low HGS contributed to the increased risk of NAFLD, and this risk was found to be further elevated when low SES and low HGS were combined. Together, our data suggest that low SES plus low HGS is significantly associated with an increased risk of NAFLD in middle-aged Korean adults.

NAFLD is a common metabolic disorder caused by the accumulation of excess fat in the liver cells, regardless of alcohol intake. A wide variety of risk factors of NAFLD have been identified, such as age, diabetes, hypertension, and hyperlipidemia [27,28]. Recently, growing evidence has shown that SES is also one of the factors influencing the prevalence of NAFLD [6,7]. SES is defined as the position of an individual on a social–economic scale that measures a combination of education, income, occupation, place of residence, heritage, and religion [29,30,31]. It has been established that SES is linked to a wide range of health problems [32,33,34]. Specifically, SES has been shown to be associated with the major risk factors of NAFLD, including insulin resistance, obesity, and lipid metabolic disorder [6,35]. Goodman et al. reported that subjects with a lower SES have a higher BMI and increased insulin resistance compared to subjects with a higher SES [36]. Consistent with these observations, our data showed that the low-SES group had subjects with higher BMI and WC and greater proportions of smokers, physically inactive subjects, and those with hypertension and diabetes compared to the high-SES group (Table 2). Subjects with a low SES also exhibited higher FBG, TG, AST, and ALT levels than subjects with a high SES (Table 2). SES disparities appear to affect the risk factors of developing NAFLD. Indeed, we found that NAFLD indices evaluated by HSI and CNS were significantly higher in subjects with a low SES compared to the subjects with middle and high SES (Table 2). These results indicate that SES disparities are associated with the incidence of NAFLD, which is likely attributable to higher levels of risk factors.

As skeletal muscle is one of the largest and most metabolically active tissues, and it largely dictates whole-body energy metabolism and insulin sensitivity [15,37], the levels of muscle strength have also been shown to be associated with the independent risk factors of NAFLD [15,16,17]. Specifically, HGS has been proposed as an indicator of overall muscle mass and strength [38,39]. HGS is a simple, noninvasive marker of muscle strength of the upper extremities that correlates with other muscle function tests, such as knee extension strength [40]. Thus, HGS has been widely used to assess the relationship between muscle strength and the prevalence of NAFLD [18,41]. We observed that the low-HGS group had subjects with a higher BMI and WC and greater proportions of physically inactive subjects and those with hypertension, diabetes, and menopause compared to the high-HGS group (Table 3). The subjects with a low HGS also exhibited higher FBG, TG, AST, and ALT levels than subjects with a high HGS (Table 3). These data indicate that the levels of HGS may contribute to the risk factors of NAFLD. We found that NAFLD indices with HSI and CNS were significantly increased in the subjects with a low HGS compared to those with a high HGS (Table 3). Our findings suggest that HGS disparities may be associated with risk factors for NAFLD.

When the ORs for risk of NAFLD were estimated with SES and HGS, the ORs of subjects with middle and low SESs were significantly higher compared to those with a high SES (Table 4). Our findings agree with previous studies suggesting that individuals with socioeconomic deprivation are at risk of an earlier onset of NAFLD [7]. Similarly, the ORs of the subjects with middle and low HGSs were significantly higher compared to those with a high HGS (Table 4). These findings are consistent with previous studies indicating that individuals with higher HGS levels displayed significant linear decreases in the NAFLD scores [18,41]. Our observations suggest that HGS is inversely associated with the risk factors of NAFLD, and high levels of muscle strength can be one of the major approaches to prevent an increase in the risk factors of NAFLD.

It has been shown that HGS is influenced by SES components including income, education, wealth, and occupational class [21,42]. Consistent with the studies investigating the relationship between HGS and SES, our data also revealed that subjects with a high SES exhibited high levels of HGS, whereas subjects with a low SES showed low levels of HGS, leading to high NAFLD indices (Table 2 and Table 3). Based on the link between HGS and SES, we investigated the effects of the interaction between HGS and SES on NAFLD indices. We found that the ORs of subjects with a low SES and a low HGS were significantly higher for the risk of NAFLD compared to subjects with a high SES and a high HGS and a high SES and a low HGS, when the ORs for NAFLD indices were nonadjusted and adjusted for all the covariates, including age, WC, marital status, region, type of housing, smoking, regular exercise, hypertension, and menopause (Table 5). These results suggest that both a high SES and a high HGS are important factors in preventing the risk factors of NAFLD in middle-aged individuals.

The present study has several strengths. First, we used representative data to identify the effects of SES and HGS on the risk of NAFLD. Second, our study used SES based on scored income and education. Finally, we evaluated muscle strength with relative HGS, which enhances the statistical reliability of the results. The limitations of our study include the difficulty in verifying the cause–effect relationship, as this was a cross-sectional study. As NAFLD indices with HSI and CNS have a high correlation with the risk factors of NAFLD, the indices can be used to predict the risk of NAFLD. However, when patients show high levels of NAFLD indices, further examinations, including ultrasound, computed tomography (CT), magnetic resonance imaging (MRI), and a liver biopsy, are required to diagnose NAFLD.

## 5. Conclusions

The present study provides a link between SES disparities and an increase in the risk of NAFLD, showing that SES can adversely affect the major risk factors of NAFLD. Our study also demonstrates that HGS is inversely associated with the risk of NAFLD in middle-aged individuals, suggesting that muscular strength is an important parameter to predict the increased risk of NAFLD, and enhanced muscular strength may contribute to the prevention of NAFLD. Importantly, our evidence is the first to show the effects of the interaction between SES and HGS on NAFLD, indicating that a low SES combined with low HGS levels may be associated with an increased risk of NAFLD.

## Figures and Tables

**Table 1:** Characteristics of the study participants.

Variables	Total (*n* = 5272)	Men (*n* = 1678)	Women (*n* = 3594)	*p* Value
**Anthropometrics**				
Age (years)	57.1 ± 4.2	57.3 ± 4.2	57.0 ± 4.2	0.081
BMI (kg/m^2^)	24.1 ± 3.2	24.4 ± 2.8	24.0 ± 3.3	<0.001
WC (cm)	82.2 ± 8.9	86.4 ± 7.8	80.3 ± 8.7	<0.001
Absolute HGS (kg)	29.7 ± 9.1	40.6 ± 6.5	24.6 ± 4.4	<0.001
Relative HGS (kg/BMI)	47.8 ± 11.8	1.68 ± 0.30	1.04 ± 0.22	<0.001
**Socioeconomic status**				
Household income (KRW 10,000/month)	449.7 ± 330.8	473.3 ± 322.5	438.6 ± 334.2	<0.001
Education (years)	11.4 ± 3.8	12.5 ± 3.8	10.9 ± 3.7	<0.001
**Sociodemographic status**				
Marital status, *n* (%)				<0.001
Married	4487 (85.1)	1501 (89.5)	2986 (83.1)	
Widow/divorced	686 (13.0)	123 (7.3)	563 (15.6)	
Unmarried	99 (1.9)	54 (3.2)	45 (1.3)	
Region, *n* (%)				0.009
Urban	4290 (81.4)	1331 (79.3)	2959 (82.3)	
Rural	982 (18.6)	347 (20.7)	635 (17.7)	
Type of housing, *n* (%)				0.263
Apartment	2762 (52.4)	898 (53.5)	1864 (51.9)	
General house	2510 (47.6)	780 (46.5)	1730 (48.1)	
**Health-related factors**				
Smoking, *n* (%)	1494 (28.3)	1304 (77.7)	190 (5.3)	<0.001
Alcohol consumption, *n* (%)	138 (2.6)	65 (3.9)	73 (2.0)	<0.001
Regular exercise, *n* (%)	1115 (21.1)	444 (26.5)	671 (18.7)	<0.001
Hypertension, *n* (%)	1304 (24.7)	461 (27.5)	843 (23.5)	0.002
Diabetes, *n* (%)	512 (9.7)	217 (12.9)	295 (8.2)	<0.001
Menopause, *n* (%)	3202 (60.7)	0 (0.0)	3202 (89.1)	<0.001

Note: BMI: body mass index, WC: waist circumference, HGS: handgrip strength.

**Table 2:** Descriptive statistics of the measured parameters according to SES categories.

Variables	Low SES (*n* = 1247)	Middle SES (*n* = 2646)	High SES (*n* = 1379)	*p* for Linear Trends
**Socioeconomic status**				
Household income (KRW 10,000/month)	157.7 ± 134.9	404.3 ± 237.3	800.6 ± 299.6	<0.001
Education (years)	7.6 ± 2.9	11.3 ± 2.7	15.1 ± 2.7	<0.001
SES index	15.3 ± 6.5	41.0 ± 11.2	73.0 ± 12.6	<0.001
**Anthropometrics**				
Women, *n* (%)	817 (65.5)	1780 (67.3)	997 (72.3)	<0.001
Age (years)	59.0 ± 3.8	57.0 ± 4.1	55.6 ± 3.9	<0.001
BMI (kg/m^2^)	24.6 ± 3.4	24.2 ± 3.1	23.4 ± 3.0	<0.001
WC (cm)	84.0 ± 9.2	82.5 ± 8.8	80.0 ± 8.5	<0.001
Absolute HGS (kg)	29.1 ± 9.2	30.2 ± 9.3	29.3 ± 8.4	0.685
Relative HGS (kg/BMI)	1.20 ± 0.41	1.26 ± 0.40	1.26 ± 0.35	<0.001
**Sociodemographic status**				
Marital status, *n* (%)				<0.001
Married	886 (71.1)	2294 (86.7)	1307 (94.7)	
Widow/divorced	308 (24.6)	315 (11.9)	63 (4.6)	
Unmarried	53 (4.3)	37 (1.4)	9 (0.7)	
Region, *n* (%)				<0.001
Urban	911 (73.1)	2142 (81.0)	1237 (89.7)	
Rural	336 (26.9)	504 (19.0)	142 (10.3)	
Type of housing, *n* (%)				<0.001
Apartment	430 (34.5)	1295 (48.9)	1037 (75.2)	
General house	817 (65.5)	1,351 (51.1)	342 (24.8)	
**Health-related factors**				
Smoking, *n* (%)	421 (33.8)	771 (29.1)	302 (21.9)	<0.001
Alcohol consumption, *n* (%)	42 (3.4)	61 (2.3)	35 (2.5)	0.201
Regular exercise, *n* (%)	139 (11.1)	541 (20.4)	435 (31.5)	<0.001
Hypertension, *n* (%)	428 (34.3)	636 (24.0)	240 (17.4)	<0.001
Diabetes, *n* (%)	172 (13.8)	257 (9.7)	83 (6.0)	<0.001
Menopause, *n* (%)	778 (62.4)	1,607 (60.7)	817 (59.2)	0.100
**Blood markers**				
FBG (mg/dL)	106.2 ± 28.0	102.2 ± 23.9	99.2 ± 18.0	<0.001
HDL-C (mg/dL)	49.1 ± 11.8	50.6 ± 12.3	52.5 ± 12.8	<0.001
TG (mg/dL)	149.5 ± 105.7	133.8 ± 85.2	126.8 ± 89.4	<0.001
AST (IU/L)	24.5 ± 12.0	23.4 ± 8.8	22.9 ± 8.5	<0.001
ALT (IU/L)	23.5 ± 15.5	22.7 ± 14.3	22.0 ± 13.8	0.011
Uric acid (mg/dL)	5.79 ± 0.85	5.84 ± 0.87	5.82 ± 0.84	0.492
**NAFLD index**				
HSI	33.8 ± 4.8	33.3 ± 4.5	32.5 ± 4.4	<0.001
CNS	58.4 ± 31.4	52.3 ± 31.3	44.2 ± 31.1	<0.001

Note: SES: socioeconomic status, BMI: body mass index, WC: waist circumference, HGS: handgrip strength, FBG: fasting blood glucose, HDL-C: high-density lipoprotein cholesterol, TG: triglyceride, AST: aspartate aminotransferase, ALT: alanine aminotransferase, NAFLD: non-alcoholic fatty liver disease, HSI: hepatic steatosis index, CNS: comprehensive NAFLD score.

**Table 3:** Descriptive statistics of the measured parameters according to the relative HGS categories.

Variables	Low HGS (*n* = 1317)	Middle HGS (*n* = 2638)	High HGS (*n* = 1317)	*p* for Linear Trends
**Handgrip strength**				
Absolute HGS (kg)	24.5 ± 7.7	29.9 ± 8.2	34.4 ± 9.3	<0.001
Relative HGS (kg/BMI)	0.94 ± 0.28	1.24 ± 0.31	1.56 ± 0.37	<0.001
**Socioeconomic status**				
Household income (KRW 10,000/month)	408.3 ± 33.41	451.7 ± 326.0	486.9 ± 332.8	<0.001
Education (years)	10.7 ± 4.1	11.5 ± 3.8	12.0 ± 3.4	<0.001
SES index	38.6 ± 23.9	43.7 ± 22.8	47.1 ± 21.9	<0.001
**Anthropometrics**				
Women, *n* (%)	898 (68.2)	1798 (68.2)	898 (68.2)	1.000
Age (years)	57.9 ± 4.2	57.2 ± 4.1	56.1 ± 4.1	<0.001
BMI (kg/m^2^)	26.2 ± 3.4	24.1 ± 2.7	22.1 ± 2.3	<0.001
WC (cm)	87.4 ± 9.2	82.0 ± 7.9	77.4 ± 7.5	<0.001
**Sociodemographic status**				
Marital status, *n* (%)				<0.001
Married	1063 (80.7)	2248 (85.2)	1176 (89.3)	
Widow/divorced	215 (16.3)	343 (13.0)	128 (9.7)	
Unmarried	39 (3.0)	47 (1.8)	13 (1.0)	
Region, *n* (%)				0.109
Urban	1050 (79.7)	2158 (81.8)	1082 (82.2)	
Rural	267 (20.3)	480 (18.2)	235 (17.8)	
Type of housing, *n* (%)				0.001
Apartment	652 (49.5)	1370 (51.9)	740 (56.2)	
General house	665 (50.5)	1268 (48.1)	577 (43.8)	
**Health-related factors**				
Smoking, *n* (%)	374 (28.4)	746 (28.3)	374 (28.4)	0.995
Alcohol consumption, *n* (%)	29 (2.2)	66 (2.5)	43 (3.3)	0.088
Regular exercise, *n* (%)	207 (15.7)	578 (21.9)	330 (25.1)	<0.001
Hypertension, *n* (%)	431 (32.7)	661 (25.1)	212 (16.1)	<0.001
Diabetes, *n* (%)	183 (13.9)	248 (9.4)	81 (6.2)	<0.001
Menopause, *n* (%)	813 (61.7)	1631 (61.8)	758 (57.6)	0.028
**Blood markers**				
FBG (mg/dL)	106.9 ± 30.3	102.2 ± 21.9	98.2 ± 18.3	<0.001
HDL-C (mg/dL)	48.5 ± 11.9	50.7 ± 12.2	52.9 ± 12.9	<0.001
TG (mg/dL)	149.1 ± 94.8	138.2 ± 98.4	117.2 ± 69.9	<0.001
AST (IU/L)	24.7 ± 10.4	23.3 ± 9.8	22.6 ± 8.3	<0.001
ALT (IU/L)	25.6 ± 16.5	22.7 ± 14.4	19.9 ± 11.6	<0.001
Uric acid (mg/dL)	5.73 ± 0.80	5.83 ± 0.87	5.90 ± 0.88	<0.001
**NAFLD index**				
HSI	35.9 ± 4.8	33.2 ± 4.2	30.5 ± 3.6	<0.001
CNS	69.4 ± 28.4	52.3 ± 30.2	32.5 ± 26.5	<0.001

Note: HGS: handgrip strength, SES: socioeconomic status, BMI: body mass index, WC: waist circumference, FBG: fasting blood glucose, HDL-C: high-density lipoprotein cholesterol, TG: triglyceride, AST: aspartate aminotransferase, ALT: alanine aminotransferase, NAFLD: non-alcoholic fatty liver disease, HSI: hepatic steatosis index, CNS: comprehensive NAFLD score.

**Table 4:** Odds ratio for NAFLD risk by socioeconomic status and relative handgrip strength.

	OR (95% CI)	*p* Value
**SES categories**		
High SES	1 (reference)	
Middle SES	1.340 (1.144–1.570)	<0.001
Low SES	1.703 (1.424–2.037)	<0.001
Each one score increase	1.008 (1.006–1.011)	<0.001
**Relative HGS categories**		
High HGS	1 (reference)	
Middle HGS	4.300 (3.402–5.435)	<0.001
Low HGS	12.161 (9.548–15.488)	<0.001
Each 0.1 kg/BMI increase	1.157 (1.135–1.179)	<0.001

Note: SES: socioeconomic status, HGS: handgrip strength, BMI: body mass index.

**Table 5:** Joint association of socioeconomic status and relative handgrip strength with the risk of NAFLD.

	Crude Model		Model ^1^	
	OR (95% CI)	*p* Value	OR (95% CI)	*p* Value
**High SES plus**				
High HGS	1 (reference)		1 (reference)	
Low HGS	9.286 (5.868–14.694)	<0.001	2.277 (1.307–3.967)	0.004
**Low SES plus**				
High HGS	1.364 (0.748–2.490)	0.311	0.811 (0.391–1.682)	0.573
Low HGS	13.499 (8.755–20.812)	<0.001	2.479 (1.351–4.549)	0.003

Note: SES: socioeconomic status, HGS: handgrip strength; Model ^1^: adjusted for age, WC, marital status, region, type of housing, smoking, alcohol consumption, regular exercise, hypertension, and menopause.

## Data Availability

The data presented in this study are available on request from the corresponding author.
